# IFT80 is required for stem cell proliferation, differentiation, and odontoblast polarization during tooth development

**DOI:** 10.1038/s41419-018-0951-9

**Published:** 2019-01-25

**Authors:** Xue Yuan, Xu Cao, Shuying Yang

**Affiliations:** 1 Department of Oral Biology, School of Dental Medicine University of Buffalo, State University of New York, Buffalo, NY USA; 2 Department of Orthopedic Surgery, Johns Hopkins University School of Medicine, Baltimore, MD USA; 3 Department of Anatomy and Cell Biology, School of Dental Medicine, University of Pennsylvania, Philadelphia, PA USA

## Abstract

Primary cilia and intraflagellar transport (IFT) proteins control a wide variety of processes during tissue development and homeostasis. However, their role in regulation of stem cell properties during tooth development remains elusive. Here, we revealed that dental pulp stem cells (DPSCs) express IFT80, which is required for maintaining DPSC properties. Mice with deletion of *IFT80* in odontoblast lineage show impaired molar root development and delayed incisor eruption through reduced DPSC proliferation and differentiation, and disrupted odontoblast polarization. Impaired odontoblast differentiation resulted from disrupted hedgehog (Hh) signaling pathways. Decreased DPSC proliferation is associated with impaired fibroblast growth factor 2 (FGF2) signaling caused by loss of IFT80, leading to the disruption of FGF2-FGFR1-PI3K-AKT signaling in *IFT80*-deficient DPSCs. The results provide the first evidence that IFT80 controls tooth development through influencing cell proliferation, differentiation, and polarization, and Hh and FGF/AKT signaling pathways, demonstrating that IFT proteins are likely to be the new therapeutic targets for tooth and other tissue repair and regeneration.

## Introduction

Tooth development, like many other organs’ formation, requires multi-lineage cells controlled by different signaling pathways. During tooth formation, a subpopulation of mesenchymal cells differentiates into odontoblasts, which are the columnar polarized cells located at the outer edges of dental pulp, expressing dentin matrix protein 1 (DMP1) and dentin sialophosphoprotein (DSPP), and producing dentin. Ameloblasts are derived from dental epithelium and secrete enamel matrix, which is the hardest and highly mineralized tissue. Dental pulp is the soft tissue inside the tooth, and their function is to support dentin formation and regeneration^1,2^.

Primary cilia are highly conserved microtubule-based solitary organelles that are present on almost all vertebrate cells. Formation and function of primary cilia require intraflagellar transport (IFT) proteins and other ciliary proteins^3,4^. Mutation of those proteins usually causes cilia defects, leading to a wide range of diseases called ciliopathies. These disorders target multiple tissues, of which bone and tooth are most common tissues^5–7^. The arising evidence demonstrates that primary cilia play important roles in tooth development^8,9^. For example, *Wnt1-Cre; Kif3a*^*fl/fl*^ mice (deletion of ciliary motor-*Kif3a* in dental mesenchyme) show impaired incisor and molar development^9,10^. Abnormal molar development was also found in *Evc*^*−/−*^^11^ and *IFT88*^*orpk*^ mice^7^. Hypomorphic alleles of IFT88 (*Tg737*^*orpk*^) showed increased Hh signaling and formation of supernumerary molars in the diastema of both the lower and upper jaws^12,13^. Previously, we reported that IFT80, one of the IFT complex B proteins, promotes osteogenesis^14^ and deletion of IFT80 in osteoblast precursor cells leads to decreased bone mass with impaired osteoblast differentiation through regulating Hh signaling pathway^15^. However, their role in regulation of stem cell properties and odontogenesis during tooth development remains elusive.

Fibroblast growth factors (FGFs) are growth factors that are important for bone and tooth development and regeneration^16^. There are 22 FGF proteins that interact with four highly conserved transmembrane tyrosine kinase receptors (FGFR1–4). Among FGFs, FGF2 is highly expressed in odontoblast lineage cells^17^. Several studies supported that FGF2 promotes the pulp cell proliferation, self-renewal, migration, and early differentiation^18–22^. However, the relationship between FGF signaling and primary cilia/IFT proteins has not been established.

In this study, we generated *IFT80* conditional knockout mouse by breeding *IFT80*-floxed mice with *Osterix (OSX)-Cre* transgenic mice and examined their phenotypic and molecular changes in tooth development. In addition, we determined the mechanism by which IFT80 regulates dental stem cell proliferation, differentiation, and polarization during tooth development. We found for the first time that IFT80 governs tooth development through influencing DPSC proliferation, differentiation, and odontoblast polarization by regulating Hh and FGF/AKT signaling pathways, demonstrating that IFT proteins are likely new therapeutic targets for tooth and other tissue repair and regeneration.

## Results

### Conditional deletion of IFT80 impaired incisor development

**Fig. 1:**
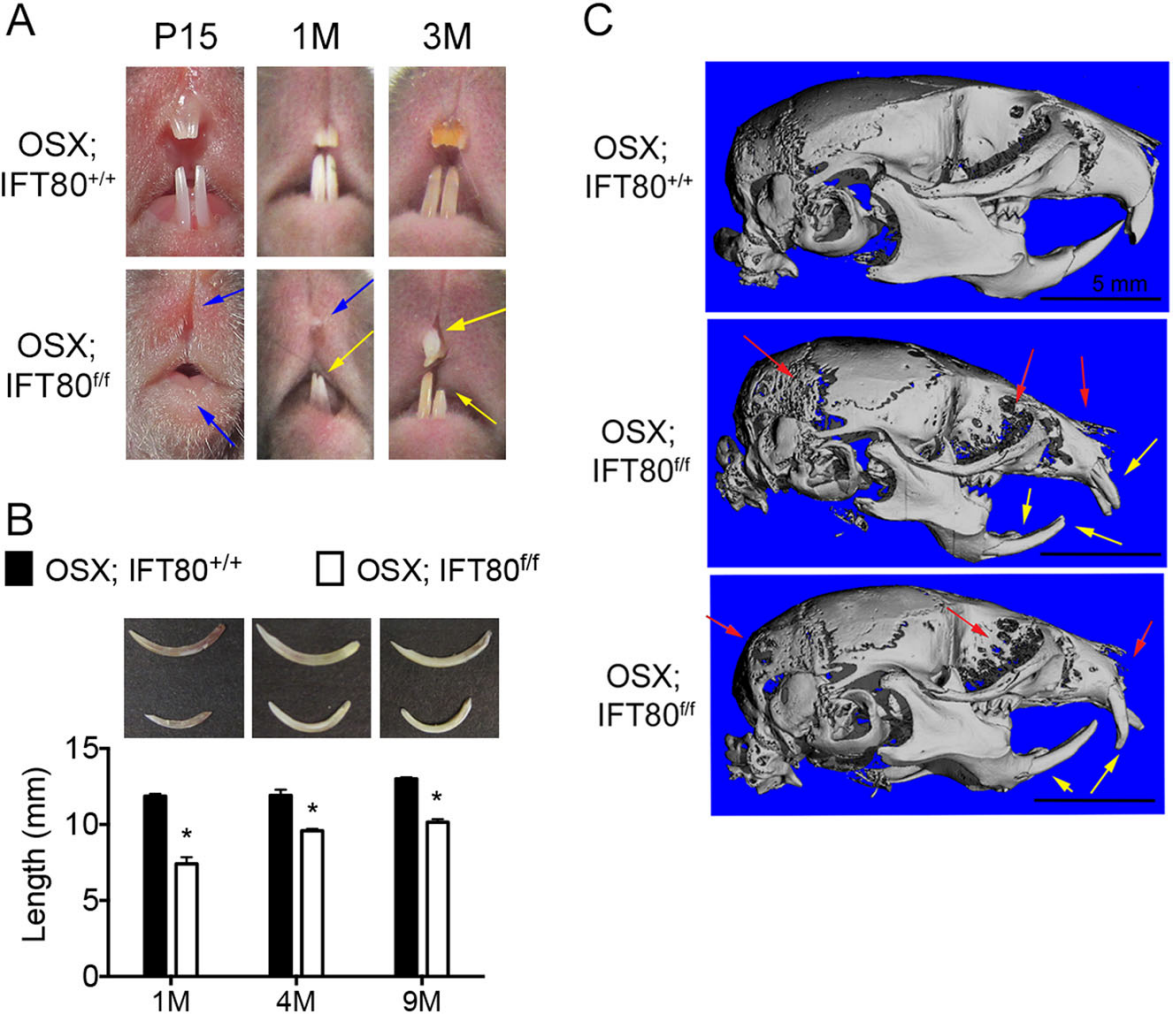
*OSX;IFT80*^*f/f*^ mice show impaired incisor eruption and development. **a** Photographic analysis of incisor development. Blue arrows indicate missing incisors. Yellow arrows indicate abnormal incisors. **b** Average length of lower incisors (*n* = 6, *p < 0.001 versus *OSX;IFT80*^*+/+*^ at different time points). **c** Side view of micro-CT to show the malocclusion (yellow arrows) and impaired craniofacial mineralization in 1M *OSX;IFT80*^*f/f*^ mice (red arrows). Scale bars represent 5 mm. Data are expressed as mean ± SEM; *p < 0.001

OSX is a transcription factor during osteoblast differentiation from stem cells and OSX+ cells are essential for bone development^23^. Recent studies demonstrate that OSX is also expressed in pulp cells during differentiation of odontoblasts^24,25^. Therefore, we generated *OSX;IFT80*^*f/f*^ mice to study the function of IFT80 in tooth development. We observed that incisors were completely absent in 15-day-old *OSX;IFT80*^*f/f*^ mice, and severely underdeveloped and malocclusioned in 1-month-old and 3-month-old *OSX;IFT80*^*f/f*^ mice (Fig. 1a). The average incisor eruption age was around postnatal day 7 in *OSX;IFT80*^*+/+*^ mice, whereas it was delayed to postnatal day 14 for lower incisors and postnatal day 21 for upper incisors in *OSX;IFT80*^*f/f*^ mice. Mandibular incisors were isolated from their sockets for morphological analysis. *OSX;IFT80*^*f/f*^ incisors were obviously shorter but more curved at all examined time points (Fig. 1b). The mean length of lower incisors in *OSX;IFT80*^*f/f*^ mice was only 0.61-fold of that in *OSX;IFT80*^*+/+*^ mice at 1 month old (Fig. 1b). Examination of skulls by micro computed tomography showed the malocclusion and defects in both mandibular and maxillary incisors in *OSX;IFT80*^*f/f*^ mice (Fig. 1c). These data suggest that IFT80 is critical for incisor development. Notably, *OSX;IFT80*^*f/f*^ mice also showed markedly decreased bone mass in craniofacial bones as well as alveolar bones (Fig. 1c).

### Conditional deletion of IFT80 impaired pulp cell proliferation in the cervical loop

**Fig. 2:**
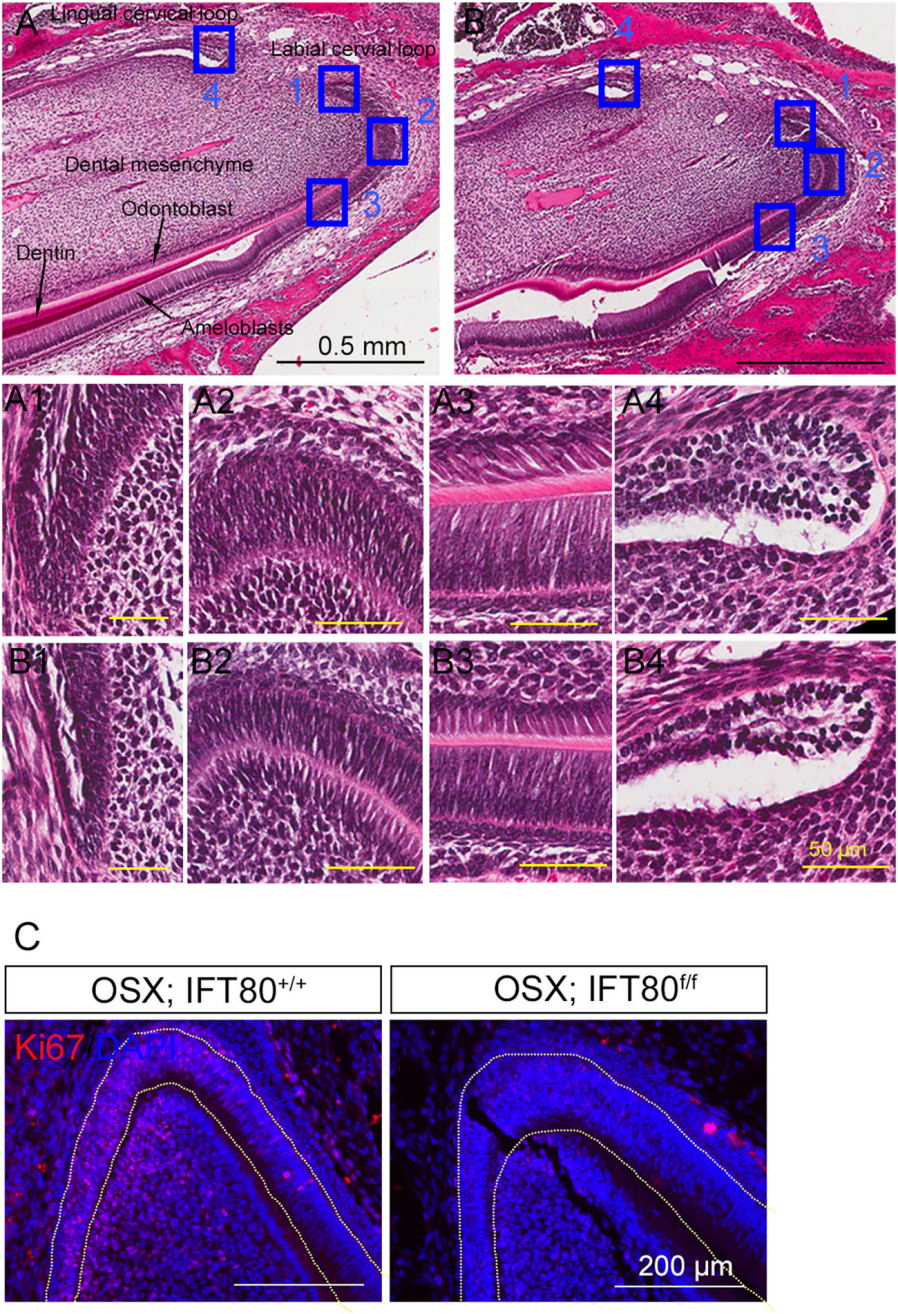
Pulp cell proliferation in the cervical loop is impaired in *OSX;IFT80*^*f/f*^ mice. **a**, **b** Hematoxylin and eosin staining of the proximal incisor region of (**a**) *OSX;IFT80*^*+/+*^ and (**b**) *OSX;IFT80*^*f/f*^ mice. A1–A4 and B1–B4 High magnification photos to show the cell layers in cervical loop as shown in **a** and **b** (blue boxes). Scale bars represent 0.5 mm (black) or 50 μm (yellow). **c** Ki67 (red) staining of cervical loop section of *OSX;IFT80*^*+/+*^ and *OSX;IFT80*^*f/f*^ mice. DAPI staining is used as a counterstain. Scale bars represent 200 μm

Histological analysis revealed that the cervical loops and odontoblast layers were smaller in *OSX;IFT80*^*f/f*^ mice compared with those in *OSX;IFT80*^*+/+*^ mice (Fig. 2a, A1–A4 and 2b, B1–B4), suggesting that the proliferation might be compromised in this area. Therefore, we performed Ki67 staining to detect cell proliferation. As we expected, the results showed that proliferating cells were significantly reduced in the cervical loop and the dental pulp in *OSX;IFT80*^*f/f*^ mice compared to *OSX*;*IFT80*^*+/+*^ control mice (Fig. 2c). Together, these data implied that IFT80 is required for the odontoblast lineage cell proliferation and incisor growth.

### Conditional deletion of IFT80 caused shorter molar root, less mineralized dentin, and disrupted odontoblast differentiation

**Fig. 3:**
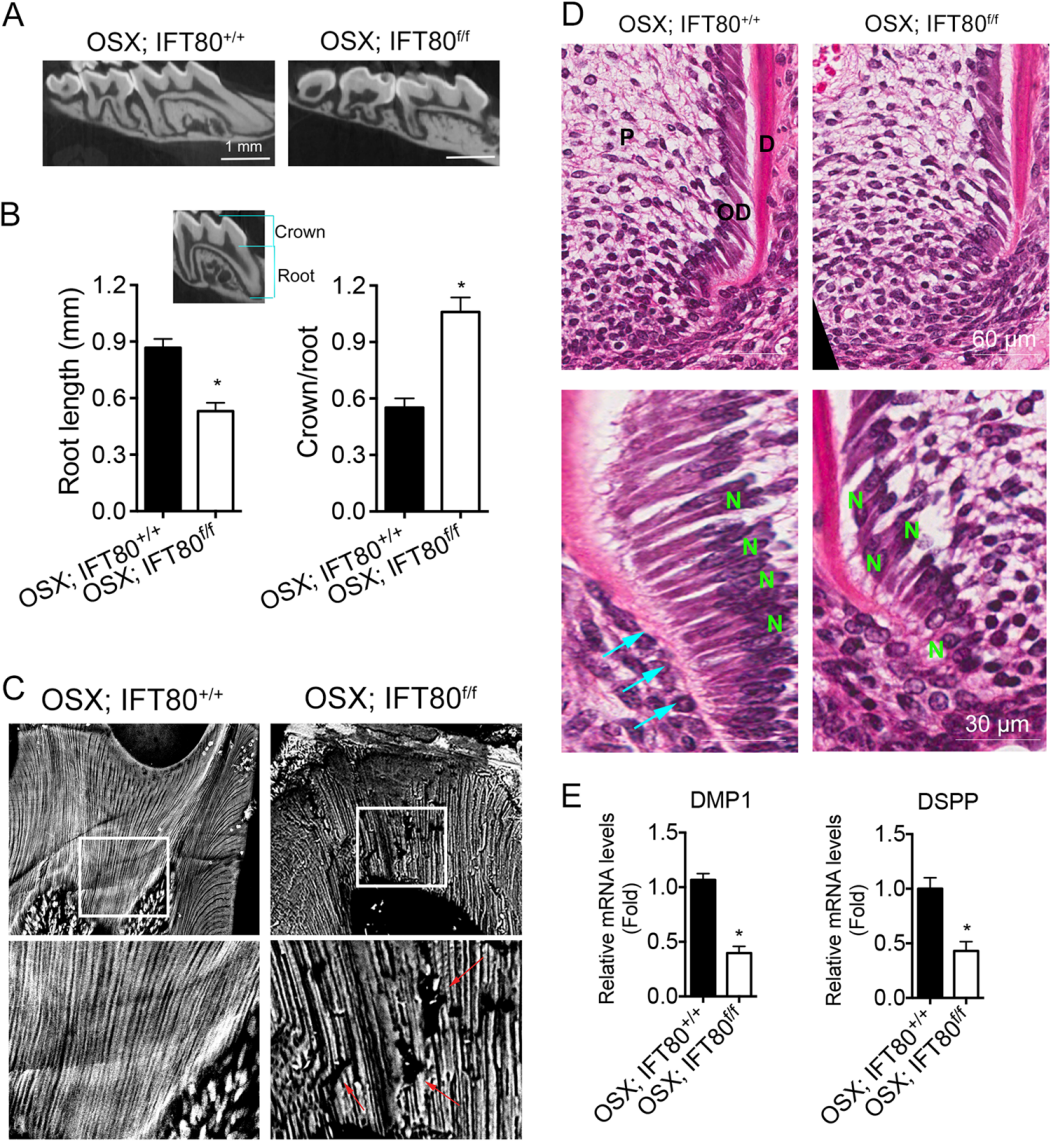
*OSX;IFT80*^*f/f*^ mice show shorter molar root, less mineralized dentin, and disrupted odontoblast differentiation. **a** Micro-tomographic view of the molar root. Scale bars represent 1 mm. **b** Measured root length and calculated crown/root ratio (*n* = 3). **c** Backscattered electron analysis of dentin tubules and mineralization. Red arrows indicate defect of dentinal tubules. Lower panels are enlarged images of the boxed areas in upper panels. **d** Hematoxylin and eosin staining of the molar root. D dentin, OD odontoblasts, P pulp, N nucleus. In *OSX;IFT80*^*+/+*^ mice, the odontoblasts were highly polarized and attached to each other by their terminal webs (cyan arrow). Lower panels are enlarged images of upper panels. Scale bars represent 60 μm (upper) or 30 μm (lower). **e** *Dentin matrix protein 1* (*DMP1*) and *dentin sialophosphoprotein* (*DSPP*) expression in the dental pulp. The expression of *DMP1* and *DSPP* was normalized to *GAPDH* expression (*n* *=* 3). Data are expressed as mean ± SEM; *p < 0.0001

We next examined molar development and found that molars were normally erupted in both *OSX;IFT80*^*f/f*^ and *OSX;IFT80*^*+/+*^ mice. The crowns of molars were well formed but the roots were shorter in *OSX;IFT80*^*f/f*^ mice compared with those in *OSX;IFT80*^*+/+*^ mice (Fig. 3a and Fig. S1A and S1B). Quantitative analysis of the root and crown length of first molars in mandible showed that the roots from *OSX;IFT80*^*f/f*^ mice were significantly shorter than those from *OSX;IFT80*^*+/+*^ mice (Fig. 3b), whereas crown length was similar in both groups. Thus, the crown-to-root ratio was significantly increased in *OSX;IFT80*^*f/f*^ mice (Fig. 3b).

To further uncover the role of IFT80 in dentin formation and mineralization, we performed backscattered electron analysis. *OSX;IFT80*^*+/+*^ mice had well-organized and patterned dentinal tubules. In contrast, the dentinal tubules in *OSX;IFT80*^*f/f*^ mice were disorganized with less mineralization (Fig. 3c). Odontoblasts are responsible for the production of calcified dentin matrix; therefore, these data suggested that IFT80 is involved in odontoblast differentiation and function. We further observed odontoblast morphology in the early stage of root formation. At postnatal day 14, odontoblasts were elongated and highly polarized in the proximal portion of the root in *OSX;IFT80*^*+/+*^ mice (Fig. 3d), and the nuclei were polarized in the pulp side of the odontoblasts. These cells were attached to each other with their terminal webs (Fig. 3d, cyan arrows). However, in *OSX;IFT80*^*f/f*^ mice, odontoblasts were disorganized along the proximal portion of the root. Some of the odontoblasts displayed a reversed cell orientation with the nuclei close to the dentin side of the odontoblasts (Fig. 3d). In addition to the morphology changes, we also found that the expression of odontoblast marker genes (*DMP1* and *DSPP*) in dental pulp was strikingly reduced in *OSX;IFT80*^*f/f*^ mice by quantitative PCR (qPCR) (Fig. 3e), confirming that *IFT80* deletion impaired odontoblast differentiation.

### Deletion of IFT80 disrupted cilia formation in odontoblasts and dental pulp cells

**Fig. 4:**
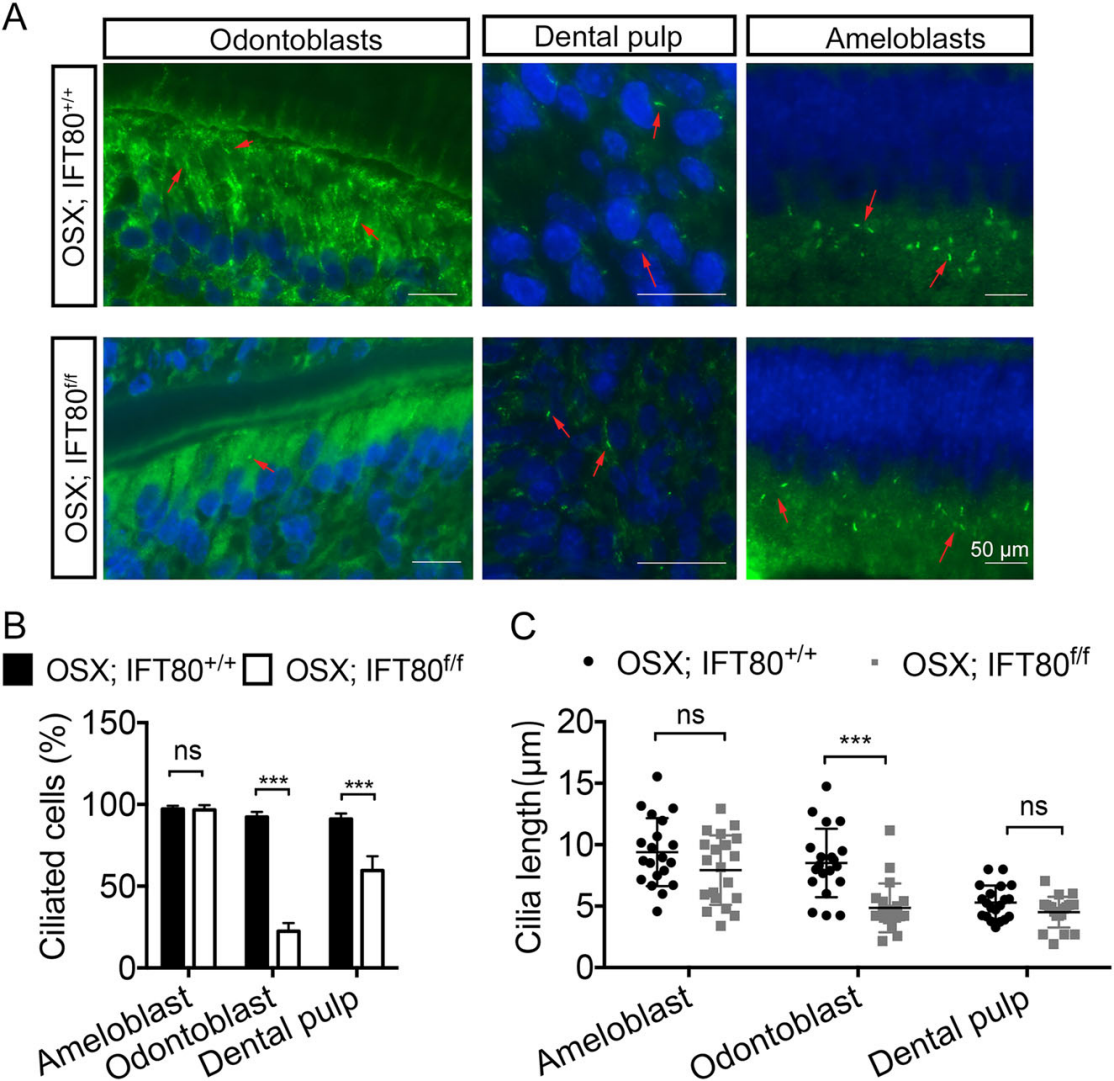
Deletion of IFT80 disrupts cilia formation and Hh signaling transduction. **a** Immunofluorescence analysis of primary cilia in molar section (red arrow). Primary cilia were stained with acetylated α-tubulin (green) antibody. DAPI is used as a counterstain. Scale bars represent 50 μm. **b** Calculated cilia percentage (*n* = 3 with at least 100 cells analyzed) is shown in **a**. **c** Calculated cilia length (*n* = 20 cells). Data are expressed as mean ± SEM; ns, not statistically significant; ***p < 0.0001

To gain insight into the mechanism that IFT80 deficiency caused defective dental pulp cell differentiation and tooth development, we first examined cilia formation (Fig. 4a). In molar section, cilia were found on almost every ameloblast and the cilia length was similar between *OSX;IFT80*^*+/+*^ mice and *OSX;IFT80*^*f/f*^ mice (Fig. 4a, b, c) However, in *OSX;IFT80*^*f/f*^ odontoblasts, cilia formation was severely disrupted. The ciliated cell population reduced to 20% in *OSX;IFT80*^*f/f*^ mice compared with 80% in *OSX;IFT80*^*+/+*^ mice (Fig. 4b). Moreover, the cilia length of ciliated odontoblasts was significantly shorter in *OSX;IFT80*^*f/f*^ mice than that in *OSX;IFT80*^*+/+*^ mice (Fig. 4c). Additionally, the ciliated cell population but not cilia length were also reduced in dental pulp cells in *OSX;IFT80*^*f/f*^ mice (Fig. 4b, c).

### Deletion of IFT80 disrupted cilia formation and Hh signaling in DPSCs

**Fig. 5:**
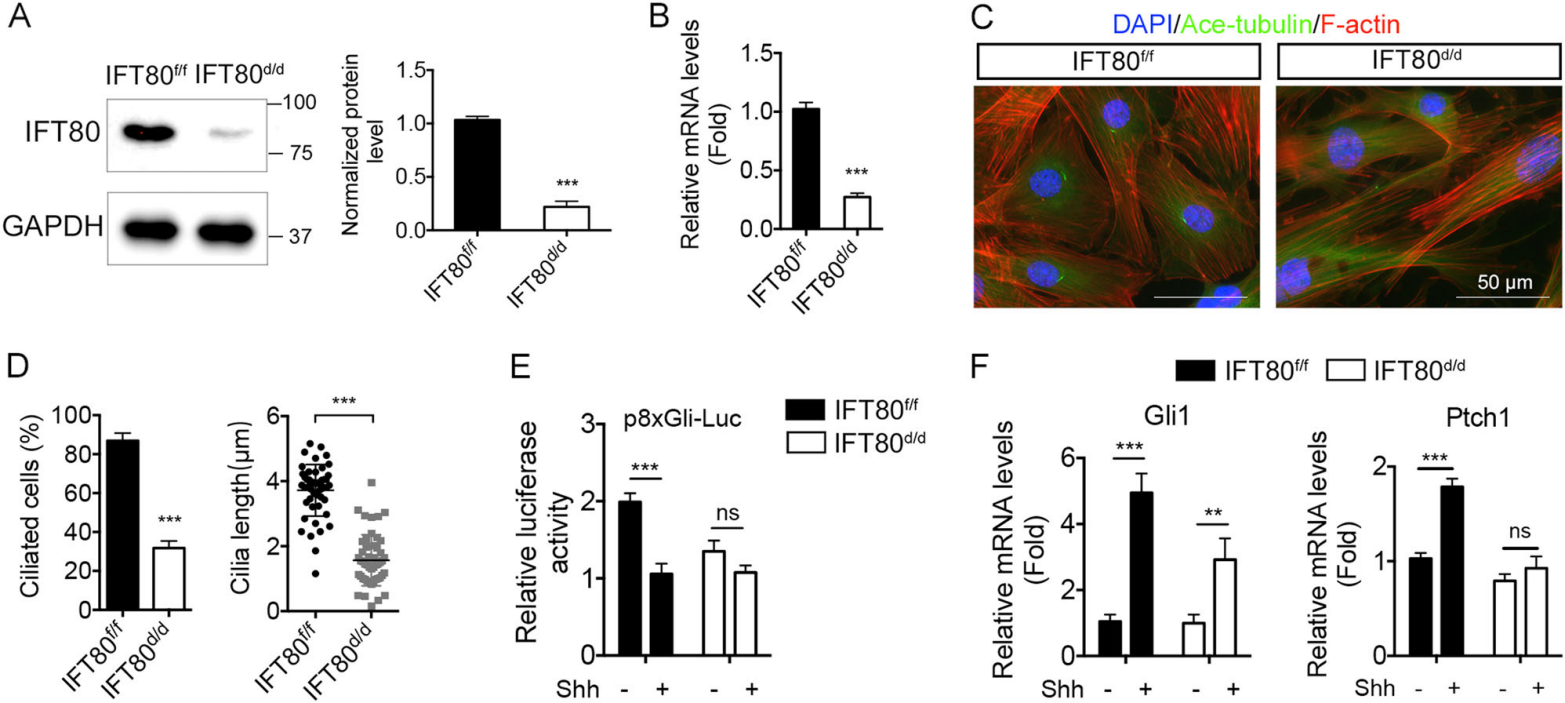
Deletion of IFT80 disrupted cilia formation as well as Hh signaling in DPSCs. **a** Western blot analysis of IFT80 expression in *IFT80*^*f/f*^ and *IFT80*^*d/d*^ DPSCs. IFT80 protein level is normalized to GAPDH (*n* = 3). **b** qPCR analysis of *IFT80* expression in *IFT80*^*f/f*^ and *IFT80*^*d/d*^ DPSCs. *IFT80* expression is normalized to *GAPDH* (*n* = 3, triplicates per group). **c** Immunofluorescence analysis of primary cilia in *IFT80*^*f/f*^ and *IFT80*^*d/d*^ DPSCs. Primary cilia were stained with acetylated α-tubulin (green) antibody. Stress fibers were stained with Alexa Fluor 568 Phalloidin (red). DAPI staining is used as counterstaining. Scale bars represent 50 μm. **d** Calculated cilia length (*n* = 20 cells) and cilia percentage (*n* = 3 with at least 200 cells analyzed). **e** Reporter assay showing 8×Gli-responsive luciferase (p8×Gli-Luc) activity in *IFT80*^*f/f*^ and *IFT80*^*d/d*^ DPSCs with or without sonic Hh (Shh. 1 μg/mL) stimulation (*n* = 3, triplicates per group). **f** qPCR results showing *Ptch1* and *Gli1* expression in *IFT80*^*f/f*^ and *IFT80*^*d/d*^ DPSCs with or without Shh stimulation (*n* = 3, triplicates per group). Data are expressed as mean ± SEM; ns, not statistically significant; ***p < 0.0001; **p < 0.001

To further examine the role of IFT80 in dental pulp cell proliferation and differentiation in vitro, we isolated primary DPSCs from the dental pulp of mouse incisor. DPSCs isolated from *IFT80*^*f/f*^ mice were infected with adenovirus-expressing Cre recombinase to delete *IFT80* (named as *IFT80*^*d/d*^). Adenovirus expressing green fluorescent protein (GFP)-infected *IFT80*^*f/f*^ DPSCs were used as control (still marked as *IFT80*^*f/f*^). Western blot and qPCR confirmed that Cre adenovirus transduction significantly reduced IFT80 expression in DPSCs (Figs. 5a, b).

We compared cilia formation between *IFT80*^*f/f*^ and *IFT80*^*d/d*^ DPSCs by immunostaining. Consistent to the results from in vivo tooth slide staining (Fig. 5a, b), the results showed that the ciliated population in *IFT80*^*d/d*^ DPSCs was reduced to <30% and the average cilia length was decreased to 1.56 μm compared with 80% and 3.72 μm in *IFT80*^*f/f*^ DPSCs, respectively (Fig. 5c, d). These results demonstrated that IFT80 is required for cilia formation in both dental pulp cells and odontoblasts. Primary cilia are essential for Hh signaling transduction, and our previous study has demonstrated that *IFT80* is required for Hh signaling transduction in osteoblast differentiation^15^; therefore, we tested Hh signaling activation in *IFT80*^*f/f*^ and *IFT80*^*d/d*^ DPSCs. As expected, sonic Hh (Shh) activated Gli promoter activity as well as *Gli1* and *Ptch1* expression in *IFT80*^*f/f*^ DPSCs (Fig. 5e, f), indicating Hh pathway activation. However, these responses were impaired in *IFT80*^*d/d*^ DPSCs (Fig. 5e, f), confirming disrupted Hh signaling transduction in *IFT80*^*d/d*^ DPSCs.

### Deletion of IFT80 impaired DPSC proliferation through defect of FGF signaling rather than Hh signaling

**Fig. 6:**
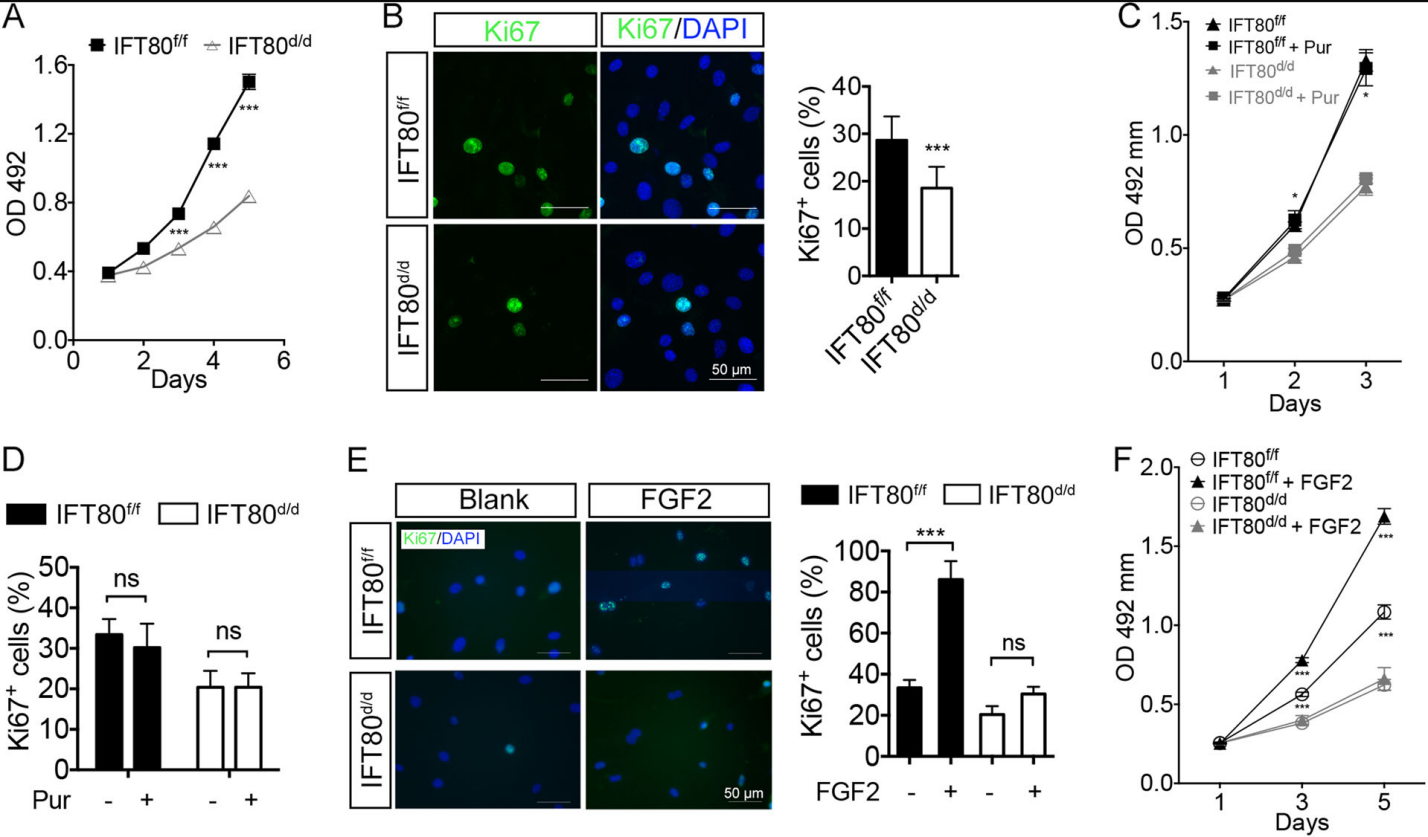
Deletion of IFT80 impaired DPSC proliferation through defect of FGF signaling rather than Hh signaling. **a** Proliferation (MTS) assay of *IFT80*^*f/f*^ and *IFT80*^*d/d*^ DPSCs. The starting cell density is 100 cells per mm^2^ (*n* = 3, triplicates per group). **b** Ki67 (green) staining of *IFT80*^*f/f*^ and *IFT80*^*d/d*^ DPSCs at the density of 500 cells per mm^2^. DAPI staining is used as counterstaining. Scale bars represent 50 μm. **c** Proliferation (MTS) assay of *IFT80*^*f/f*^ and *IFT80*^*d/d*^ DPSCs treated with or without purmorphomine (Pur, 2 μM). The starting cell density is 100 cells per mm^2^ (*n* = 3, triplicates per group). **d** Ki67-positive cell of *IFT80*^*f/f*^ and *IFT80*^*d/d*^ DPSCs treated with or without purmorphomine (Pur, 2 μM) (*n* = 3 with at least 200 cells analyzed). **e** Ki67 (green) staining of *IFT80*^*f/f*^ and *IFT80*^*d/d*^ DPSCs treated with FGF2 (10 ng/mL) at the density of 500 cells per mm^2^. DAPI staining is used as counterstaining. Scale bars represent 50 μm. **f** Proliferation (MTS) assay of *IFT80*^*f/f*^ and *IFT80*^*d/d*^ DPSCs treated with FGF2 (10 ng/mL). The starting cell density is 100 cells per mm^2^ (*n* = 3, triplicates per group). Data are expressed as mean ± SEM; ns, not statistically significant; *p < 0.05; ***p < 0.0001

Since our in vivo results suggested that *OSX;IFT80*^*f/f*^ mice has less proliferating cells in dental pulp (Fig. 2c), we then compared the proliferation rate between *IFT80*^*f/f*^ and *IFT80*^*d/d*^ DPSCs by MTS assay and Ki67 staining. The results showed that *IFT80* deletion inhibits DPSC proliferation (Fig. 6a, b). To further test if disrupted Hh signaling in *IFT80*^*d/d*^ DPSCs contributes to the DPSC proliferative defect, Hh agonist purmorphomine was used to stimulate DPCSs in both *IFT80*^*f/f*^ and *IFT80*^*d/d*^ groups. The results showed that activation of Hh signaling by purmorphomine did not affect DPSC proliferation in both groups (Fig. 6c, d), suggesting that the pathway(s) other than Hh signaling existed to regulate DPSC proliferation and were affected by loss of IFT80.

FGF signaling regulates the proliferation of many types of cells including dental pulp cells^18,26^. We found that FGF2 dramatically promotes proliferation of *IFT80*^*f/f*^ DPSCs, but failed to increase the proliferation of *IFT80*^*d/d*^ DPSCs (Fig. 6e, f), indicating that deletion of *IFT80* impaired FGF2 signaling transduction, which caused defective DPSC proliferation.

### Deletion of IFT80 inhibited DPSC proliferation via down-regulation of FGF-PI3K-AKT signaling

**Fig. 7:**
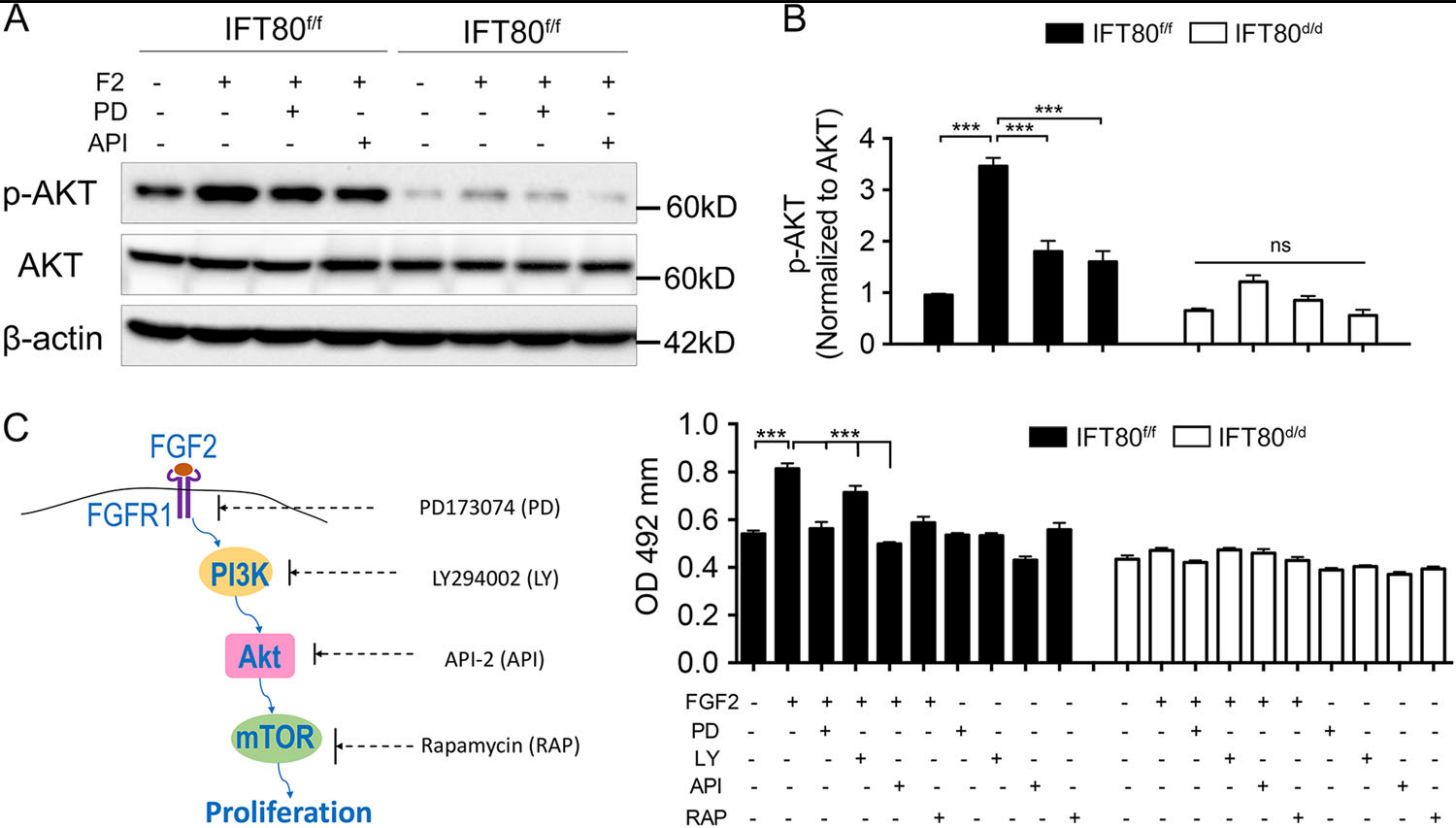
Deletion of IFT80 inhibited DPSC proliferation via down-regulation of FGF2–FGFR1–PI3K–AKT signaling. **a** Western blot analysis of AKT phosphorylation with FGF2 (10 ng/mL) stimulation in *IFT80*^*f/f*^ and *IFT80*^*d/d*^ DPSCs. AKT phosphorylation level is normalized to AKT (*n* = 3). PD PD173074 (1 μM); API API-2 (1 μM, AKT inhibitor). **b** Quantification of protein expression from **a**. AKT phosphorylation level was normalized AKT (*n* = 3). **c** Proliferation (MTS) assay of *IFT80*^*f/f*^ and *IFT80*^*d/d*^ DPSCs treated with FGF2 together with PD173074 (PD, 1 μM), LY294002 (LY, 15 μM, PI3K inhibitor), API-2 (API, 1 μM), or rapamycin (RAP, 0.5 μM, mTOR inhibitor). The starting cell density is 100 cells per mm^2^ (*n* = 3, triplicates per group). Data are expressed as mean ± SEM; ns, not statistically significant; ***p < 0.0001

Phosphorylated FGFR activates phosphatidylinositol 3-kinase (PI3K) and AKT pathway, which converts FGF signaling to the proliferative effect. Consistently, we found that, in response to FGF2 stimulation, AKT phosphorylation increased in *IFT80*^*f/f*^ DPSCs, which was inhibited by PD173074 (FGFR inhibitor) or API-2 (AKT inhibitor) treatment (Fig. 7a, b). In *IFT80*^*d/d*^ DPSCs, AKT expression was comparable to *IFT80*^*f/f*^; however, AKT phosphorylation level induced by FGF2 in *IFT80*^*d/d*^ DPSCs was much lower than that of *IFT80*^*f/f*^ DPSCs (Fig. 7a, b), confirming that FGF2-FGFR signaling was blocked in *IFT80*^*d/d*^ DPSCs. We further found that PD173074, LY294002 (PI3K inhibitor), API-2, or rapamycin (mammalian target of rapamycin (mTOR) inhibitor) inhibited the proliferation of FGF2-induced *IFT80*^*f/f*^ DPSC but not *IFT80*^*d/d*^ DPSCs (Fig. 7c). These data implied that IFT80 is essential for DPSC proliferation via regulating FGF2-FGFR1-PI3K-AKT-mTOR signaling pathway.

## Discussion

Although primary cilia have been observed in DPSCs and odontoblasts more than a decade ago^27^, the function of primary cilia and IFT proteins in DPSCs has not been well addressed. Our study reveals for the first time that IFT80 is required for tooth development by regulating DPSC proliferation, differentiation, and odontoblast polarization. Deletion of *IFT80* in DPSCs impairs FGF2-FGFR-PI3K-AKT signaling that inhibits DPSC proliferation. Moreover, *IFT80* deletion impairs cilia formation and Hh signaling transduction, which eventually inhibits DPSC differentiation and tooth development.

Primary cilium projects from the cell surface of most mammalian cells and coordinates of various signaling pathways during development and homeostasis. Although the exact roles of primary cilia in tooth development remain elusive, the emerging evidence indicates that primary cilia in both dental epithelium and mesenchyme are pivotal for early odontogenesis and later tooth development^28,29^. A most recent study showed that IFT140, one of the IFT protein that formed IFT complex A, is highly expressed in odontoblasts and knockout of IFT40 leads to delayed and defective dentin formation due to poor odontogenic differentiation, abnormal primary cilia, and decreased Hh signaling^30^. In this study, we used an *IFT80* conditional knockout mouse model, which is one of the components of IFT complex B, and found the similar dental phenotypes—reduced odontoblast proliferation and shorter root formation. Deletion of IFT140 or IFT80 all causes defects in cilia formation, suggesting that primary cilia, instead of individual IFT protein, play an essential role in the odontoblast proliferation and differentiation. However, inconsistently, hypomorphic alleles of IFT88 (*Tg737*^*orpk*^) showed increased Hh signaling and formation of supernumerary molars in the diastema of both the lower and upper jaws, indicating that IFT protein likely also regulates cilia-independent pathway during tooth development.

Hisamoto et al.^31^ studied the pattern of cilia in tooth germ and oral cavity and found that the cilia in odontoblasts first appears near the root apex and then extends along the odontoblast layer^31^. This might explain why we observed severe root formation defects in IFT80 knockout mice. Their study also indicated the stage-specific and region-specific cilia formation and cilia length regulation. A reasonable assumption is that the cell differentiation status determines the cilia length, which closely related to their sensory function^29,31^. In IFT80 knockout mice, we also observed the cilia length change in both pulp cells and odontoblasts (Fig. 4). Whether cilia length is directly associated with differentiation status needs further study. Our data here suggest that in addition to odontoblast differentiation and pulp cell proliferation (Fig. 2), odontoblast polarization (Fig. 3) requires functional cilia.

Primary cilium serves as a signaling hub and a variety of receptors and signaling proteins locate in cilia^3,32^. For example, the key components of Hh signaling including Patched1 (Ptch1), Smoothened, and Gli transcription factors are present in primary cilia^33,34^. It is well known that Hh signaling transduction requires IFT proteins and other ciliary protein^35^. In our previous study, we reported that IFT80 is essential for Hh signaling in osteoblasts, chondrocytes, and bone marrow derived mesenchymal stem cells^14,15,36,37^. Consistently, in this study, we found that *IFT80* deletion in DPSCs disrupts cilia formation and Hh signaling transduction (Figs. 4 and 5). In addition to Hh signaling, emerging studies have suggested that IFT proteins and primary cilia mediate platelet-derived growth factor signaling^38^, Notch^39^, insulin-like growth factor^40,41^, and epidermal growth factor^32^ signaling pathways. Besides these critical pathways, cilia formation is also regulated by other signaling pathways like FGF signaling. Mutation of FGFR1, a receptor of FGF signaling, in zebrafish shortens cilia^42^. Whether IFT proteins regulate FGF signaling was unknown. In our study, we found that loss of *IFT80* in DPSCs dramatically blocked FGF2-induced DPSC proliferation (Fig. 6), indicating that IFT80-mediated primary cilia formation is important for FGF2 transduction. It is still not clear how primary cilia loss effects FGF2 signaling. One possible explanation is that the receptor for FGF2 is normally located in primary cilia and loss of cilia leads to the dysregulation of FGFR. Another possible explanation is that primary cilia indirectly effect FGF2 signaling by targeting the downstream signaling, such as PI3K-AKT.

FGF2 signaling has been recognized as an important signaling that regulate both cell proliferation and cell differentiation; however, its functions are complicated and stage-dependent^18,22,43^. Our work showed that IFT protein/primary cilia regulates the FGF2 signaling, which indicated a new regulatory mechanism of FGF2 signaling during odontogenesis and tooth development. Further study regarding how IFT and cilia regulate stem cell self-renewal and differentiation is going on. Collectively, this study demonstrated for the first time that IFT80 is a critical regulator of DPSC proliferation and tooth development. IFT80 maintains cilia formation and stimulates DPSC proliferation. Thus, we revealed a novel role and mechanism of IFT80 in the regulation of DPSC and tooth development, and provided new insights for bone and tooth regenerative therapeutic design and therapy.

## Materials and methods

### Mice

All experiments performed on mice were approved by the University at Buffalo Institutional Animal Care and Use Committee. The generation of *IFT80*^*f/f*^ mice model (two LoxP sites flanking exon 6 of *IFT80*) was previously described^15^. *OSX-Cre* transgenic mice (also known as *OSX1-GFP::Cre* (Jackson #006361)) were crossed with *IFT80*^*f/f*^ mice to generate *OSX-Cre;IFT80*^*f/+*^ mice, which were crossed with *IFT80*^*f/f*^ mice to obtain *OSX-Cre;IFT80*^*f/f*^ mice (named as *OSX;IFT80*^*f/f*^). *OSX-Cre* mice were used as experimental controls (named as *OSX;IFT80*^*+/+*^).

### Reagents

Recombinant mouse Shh N-terminus (1 μg/mL, R&D Systems, Minneapolis, MN, USA) or purmorphomine (2 μM, Tocris Bioscience, 4551) was used to activate Hh signaling. FGF2 (10 ng/mL, R&D Systems, Minneapolis, MN, USA) was used to activate FGF signaling. PD173074 (1 μM, Tocris, 3044) was used to inhibit FGFR. LY294002 (15 μM, Sigma, L9908), API-2 (1 μM, Tocris, 2151), rapamycin (0.5 μM, Selleckchem, S1039) were employed to inhibit PI3K, AKT, and mTOR, respectively.

### Backscattered and resin-casted scanning electron microscopy

Extracted and cleaned molars were fixed and cut longitudinally. Acid etching was performed with 37% phosphoric acid for 5 s and followed by washing with 5.25% sodium hypochlorite for 5 min on the dentin surface as described before^44^. The specimens were dehydrated in a graded ethanol series (30%, 50%, 70%, 85%, and 100% for 15 min) and washed with hexamethyldisilizane. The samples were air dried overnight and then coated with carbon. A Hitachi SU70 scanning electron microscope (Hitachi Instruments, Schaumburg, IL, USA) was used to perform the analyses.

### Histology

Mouse mandibles were excised, fixed with 10% natural-buffered formalin (VWR International, West Chester, PA, USA), and decalcified in 10% EDTA (ethylenediaminetetraacetic acid, Thermo Fisher Scientific) for 2 weeks at 4 °C. Paraffin-embedded samples were sectioned and stained with hematoxylin and eosin.

### DPSC isolation, culture, and differentiation

The incisors were isolated from the mandibles that were dissected from 6-week-old *IFT80*^*f/f*^ mice. Whole dental pulp was gently collected from the interior of the incisor and exposed to enzymatic digestion with collagenase type I (3 mg/mL) and dispase (4 mg/mL) for 1 h at 37 °C with shaking. The digested tissues were homogenized by repetitive pipetting and the released cells were centrifuged at 200 × *g* for 10 min. The cells were cultured in α-modified Eagle’s medium (α-MEM, Life Technologies) containing 10% fetal bovine serum (FBS, Life Technologies), 2 mM l-glutamine (Life Technologies), 100 U/mL penicillin, and 100 μg/mL streptomycin (Life Technologies). DPSCs could adhere to the plastic dish for 24 h and then the medium was changed to remove floating debris. Culture medium was replaced every 3 days until the cells reach 80% confluence. Then, the cells were detached by 0.25% Trypsin-EDTA (Life Technologies) and sub-cultivated at a ratio of 1:2^45,46^.

DPSCs from *IFT80*^*f/f*^ mice were infected with adenovirus that overexpress either Cre (Ad-CMV-Cre, #1405, Vector Biolabs) or GFP (Ad-GFP, #1060, Vector Biolabs). Ad-CMV-Cre infection causes *IFT80* deletion in *IFT80*^*f/f*^ DPSCs, which were then marked as *IFT80*^*d/d*^. Ad-GFP were used as an infection control and Ad-GFP-treated *IFT80*^*f/f*^ DPSCs were still marked as *IFT80*^*f/f*^.

### Western blot

Cells were harvested and homogenized with radioimmunoprecipitation assay buffer. Proteins were denatured in sodium dodecyl sulfate (SDS) buffer and separated with SDS-polyacrylamide gel electrophoresis gels. Proteins were transferred to polyvinylidene difluoride membranes and then blocked with 5% skimmed milk (OXOID). Membranes were incubated with primary antibody overnight at 4 °C, and then incubated with horseradish peroxidase (HRP)-conjugated goat anti-rabbit immunoglobulin G (IgG) antibody (1:10,000, Novex, Carlsbad, CA, USA) at room temperature for 1 h. Visualization was performed with WesternBright ECL HRP (Bio-Rad). β-Actin (1:500, Santa Cruz) was used as the internal control.

The same procedure was used to determine the IFT80 (1:400, PAB15842, Abnova), AKT (1:300, sc-8312, Santa Cruz), and p-AKT (1:300, sc-7985-r, Santa Cruz).

### Quantitative PCR

Total RNA was extracted from cultured DPSCs with Trizol reagent (Invitrogen, Carlsbad, CA, USA) and then synthesized to complementary DNA (cDNA) with total RNA by RNA to cDNA EcoDry Premix Kit (Clontech, Palo Alto, CA, USA). qPCR was performed with ABI PRISM 7500 Real-Time PCR Machine (Invitrogen, Carlsbad, CA, USA) and SYBR Green PCR Master Mix (Invitrogen). Sequence and product length for each primer pair were listed in Supplementary Table S1. Gene expression was normalized to the housekeeping gene *GAPDH* and calculated according to the 2^−ddCT^ method^47^. All reactions were run in triplicate.

### Immunocytochemistry and immunofluorescence

Deparaffinized sections or fixed cells were permeabilized, blocked, and incubated with primary anti-Ki67 antibody (1:50 for slides, 1:200 for cells, ab16667) overnight at 4 °C. The slides were washed and stained with Alexa Fluor 568-conjugated anti-rabbit IgG (1:1000, Invitrogen) antibody or for 1 h at room temperature. DAPI (6-diamidino-2-phenylindole, Sigma) was used as a counterstain for nuclei .

The same staining procedure was used for acetylated α-tubulin (1:500, T6793, Sigma). For primary cilia staining of DPSCs, the cells were serum starved for 48 h to induce ciliogenesis.

### MTS assay

The MTS assay was performed using the CellTiter 96^®^ AQueous One Solution Cell Proliferation Assay Kit (Promega).

### Reporter assay

To test Gli promoter activity, reporter assay was performed with Gli-responsive luciferase reporter construct (8×Gli-Luc) (gift from Dr. Fernandez-Zapico^48^). DPSCs (1×10^6^) were transfected with 3 μg 8×Gli-Luc and 0.6 µg pRL-TK Renilla luciferase (Promega, internal control) with Fugene HD transfection reagent. Cells were induced with OS medium for 3 days and then stimulated with 1 μg/mL of recombinant mouse Shh N-terminus (R&D Systems, Minneapolis, MN, USA) for 8 h. Cells were harvested and colored by the Dual-luciferase Assay Kit (Promega, Madison, WI, USA) and the relative luciferase activity was measured with the Veritas microplate luminometer (Turner Biosystem, Sunnyvale, CA, USA). The experiment was conducted in triplicate.

### Statistical analysis

All data are presented as mean ± SEM (*n* = 3 or more as indicated in figure legends). Comparisons between two groups were performed by Student’s *t* test and comparisons among grouped samples were analyzed by two-way analysis of variance followed by Tukey’s multiple comparison. *P* < 0.05 was considered to be statistically significant. The program GraphPad Prism (GraphPad Software Inc., San Diego, CA, USA) was used for these analyses.

## Electronic supplementary material

Supplemental information
