# Supplementary material for: IFT80 is required for stem cell proliferation, differentiation, and odontoblast polarization during tooth development
Source: Cell Death Dis. 2019 Jan 25;10(2):63. doi: 10.1038/s41419-018-0951-9 (PMC6347632; doi:10.1038/s41419-018-0951-9)
Supplement: Supplementary file 1 — Supplemental information [file 41419_2018_951_MOESM1_ESM.docx]

Supplemental information


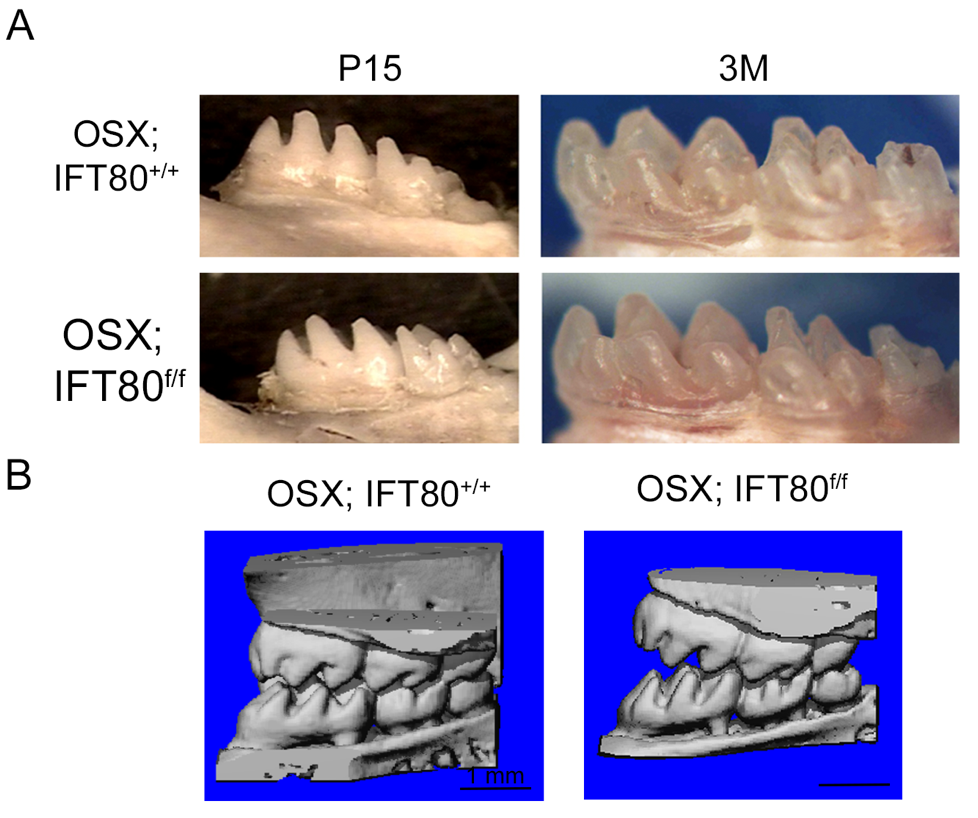


Figure S1. *OSX; IFT80^f/f^* mice show normal molar crown development. (A) Stereoscopic appearance of the molars from *OSX; IFT80^+/+^* and *OSX; IFT80^f/f^* mice at P15 and 3M. (B) Micro-tomographic view of the molar from *OSX; IFT80^+/+^* and *OSX; IFT80^f/f^* mice at 3M mice.

Supplementary table 1. List of primers used in this study.

| Gene | Forward primer sequence | Reverse primer sequence | Length |
| --- | --- | --- | --- |
| IFT80 | AAGGAACCAAAGCATCAAGAATTAG | AGATGTCATCAGGCAGCTTGAC | 148 bp |
| Gli1 | GGTCTCGGGGTCTCAAACTG | CCATTCTCTGGTGGGGTTCC | 184 bp |
| Ptch1 | GACCGGCCTTGCCTCAACCC | CAGGGCGTGAGCGCTGACAA | 204 bp |
| DMP1 | GCTTCAGGCTCAGTTTTGCT | TGTAACCCTCCAGCTCCAGG | 258 bp |
| DSPP | GGCCAATCTCATGGGGGAAA | GAGCTTTTGGTTGTCCTGCG | 177 bp |
| GAPDH | TGTGTCCGTCGTGGATCTGA | TTGCTGTTGAAGTCGCAGGAG | 150 bp |
